# *Sirt1* protects against hippocampal atrophy and its induced cognitive impairment in middle-aged mice

**DOI:** 10.1186/s12868-022-00718-8

**Published:** 2022-06-06

**Authors:** Zuhao Sun, Shuang Zhao, Xinjun Suo, Yan Dou

**Affiliations:** 1 Department of Radiology and Tianjin Key Laboratory of Functional Imaging, Tianjin Medical University General Hospital, Tianjin, 300052 People’s Republic of China; 2 School of Medical Technology, Tianjin Medical University, Tianjin, 300070 People’s Republic of China

**Keywords:** *Sirt1*, Aging, Hippocampal atrophy, Brain structural MRI, Learning and memory

## Abstract

**Background:**

*Sirtuin 1* (*Sirt1*) is a recognized longevity gene and has been shown to be associated with aging and its related diseases. Hippocampal volume is considered to be the most sensitive brain imaging phenotype for cognition, but the effect of *Sirt1* on hippocampal morphology during aging has not been reported.

**Results:**

Herein, we investigated the effect of conditional *Sirt1* knockdown on hippocampal volume in middle-aged mice, as well as its cognitive function and the underlying molecular mechanisms. Brain structural magnetic resonance imaging (MRI) showed that adeno-associated virus (AAV) mediated hippocampal *Sirt1* knockdown caused hippocampal atrophy in 8-month-old mice. Open field test (OFT) and Morris Water Maze (MWM) test revealed that hippocampal *Sirt1* knockdown significantly weakened spatial learning and memory of mice without effect on anxiety and exploratory behavior. Western blotting analysis showed that P-tau levels at serine 396 epitope were significantly increased with slightly decreased T-tau levels, while PSD95 and NMDAR2B levels were obviously reduced, indicating that hippocampal *Sirt1* knockdown could activate tau hyperphosphorylation and synaptic damage.

**Conclusions:**

This work revealed that *Sirt1* is an important protective gene against hippocampal atrophy and its induced cognitive impairment during aging, providing potential therapeutic targets for the prevention and intervention of aging-related neuropsychic diseases.

**Supplementary information:**

The online version contains supplementary material available at 10.1186/s12868-022-00718-8.

## Introduction

Aging is an important social problem facing all countries in the world today. One of the neurodegenerative diseases highly related to aging is Alzheimer’s disease (AD), whose incidence increases with age [1, 2]. The main clinical manifestations of AD are cognitive and memory impairment, accompanied by atrophy in hippocampus and other related brain areas [3–6] Its recognized pathological features mainly include β-amyloid plaques, neurofibrillary tangles and neuronal death [4]. The *2020 world AD report* suggested that brain volume atrophy occur much earlier than clinically observed symptoms of cognitive dysfunction [7]. Moreover, synaptic loss has been confirmed to be closely associated with the progression of cognitive impairment, often preceding neurodegenerative changes in above-described pathological features [8–10]. Therefore, it is of great significance to find effective neuroprotective intervention targets for delaying hippocampal atrophy and synaptic damage during aging and preventing AD.

Epigenetic regulation, such as histone post-translational modification and DNA methylation, has recently been revealed to play an important role in maintaining normal brain function, which can stabilize gene expression patterns in the brain and be crucial for long-term memory storage of information [11–13]. Histone deacetylation is a common type of histone post-translational modification [14]. One of the key factors affecting histone deacetylation is histone deacetyltransferase (HDACs), which consists of four classes (Class I, II, III and IV), and Class III HDAC is the sirtuin family [15]. The sirtuin family is a highly conserved class of HDACs that plays multiple functions in aging, chromatin integrity, metabolic regulation and longevity [16]. Sirtuin 1 (SIRT1), the most widely studied gene, is mainly expressed in neurons and has been reported to play a key role in regulating nerve progenitor cell fate, axonal dendritic differentiation and synaptic plasticity [17].

Changes in *Sirt1* expression have been proved to be closely related to the progression of cognitive impairment and AD pathology. Studies have shown that *Sirt1* overexpression in the hippocampus can induce cognitive enhancement in both 3xTg-AD model mice and healthy non-transgenic mice [18]. Furthermore, *Sirt1* overexpression in tauopathy mouse models could enhance the activation of ubiquitin-proteasome system (UPS) and effective clearance of phosphorylated tau protein (P-tau) [19, 20]. In *Sirt1* knockout mice, the brain morphology and dendritic spine structure were similar to those of healthy mice, but the complexity of synaptic network was reduced and synaptic plasticity was weakened [21, 22]. In conclusion, *Sirt1* gene has an obvious neuroprotective effect, and exploring its protective effect on cognitive function of healthy aged mice is of great significance for the prevention of cognitive disorders such as AD.

Herein, we studied the neuroprotective effect of *Sirt1* gene on hippocampal volume and cognitive function in middle-aged mice. First, *Sirt1* interference plasmid was constructed and packaged with lentivirus to verify its knockdown efficiency in mouse glioma cells. Then, the plasmid with the best knockdown efficiency was packaged as adeno-associated virus (AAV) and injected into the dorsal hippocampal CA1 region of 8-month-old C57/BL mice, compared with no-load AAV injection. After three weeks, 3T brain structural magnetic resonance imaging (MRI) was used to detect the hippocampal volume, and then open field test (OFT) and Morris water maze (MWM) test were performed to assess the learning and memory ability of mice. Finally, hippocampal tissues were taken out for western blotting to evaluate the changes of tau-related proteins (T-tau, P-tau at Ser396) as well as synaptic proteins (PSD95, Synaptophysin, Synapsin1, and NMDAR2B). This work revealed that *Sirt1* is an important protective gene in maintaining hippocampal volume and cognitive function during aging, providing potential therapeutic targets for the prevention and intervention of aging-related diseases such as AD.

## Results

### *Sirt1* shRNA knockdown efficiency in vitro and in vivo

**Fig. 1:**
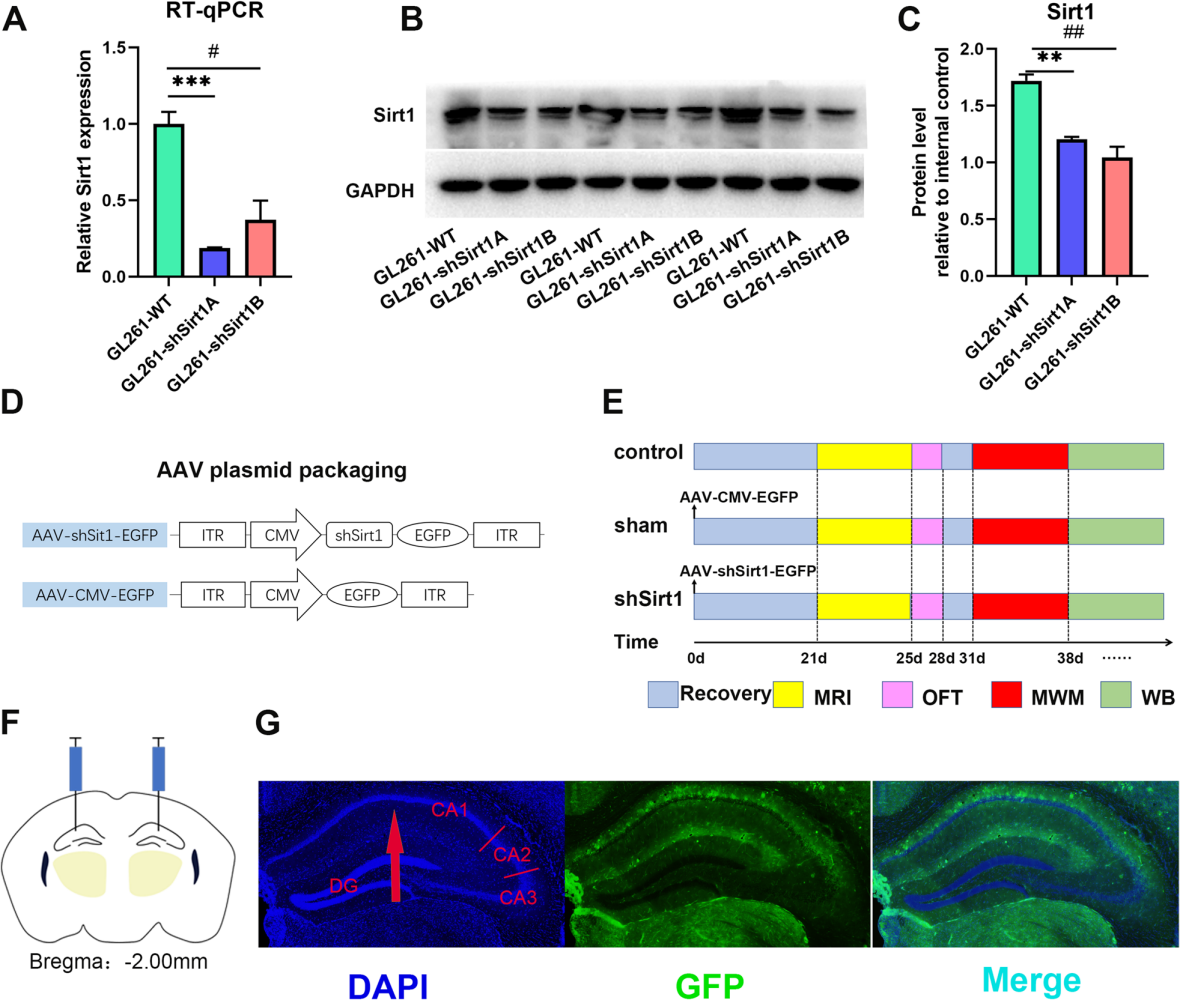
Design of shRNA targeting ***Sirt1*** for AAV packaging and hippocampal *Sirt1* knockdown by stereotactic injection.** A** Validation of knockdown efficiency of *Sirt1*-shRNAs in GL261 cells by RT-qPCR. Abbreviations: GL261-WT (wide type GL261 cells), GL261-shSirt1A (GL261 cells infected by the first shRNA sequence targeting *Sirt1*), GL261-shSirt1B (GL261 cells infected by the second shRNA sequence targeting *Sirt1*). n = 3. ^***^p < 0.001 represents comparison between the GL261-WT and GL261-shSirt1A. ^#^*p* < 0.05 represents comparison between the GL261-WT and GL261-shSirt1B. **B** Western blotting of *Sirt1* levels after *Sirt1* knockdown in G1261 cells. **(C)** Quantitative density values in **B**. **D** Illustration of plasmid construction for AAV packaging. Abbreviations: inverted terminal repeats (ITR), cytomegalovirus promoter (CMV), *Sirt1* shRNA (shSirt1), and enhanced green fluorescent protein (EGFP) reporter gene. **E** Work flow of in vivo experiments performed on the control group, the sham group and the shSirt1 group. Abbreviations: Open Field test (OFT), Morris Water Maze (MWM), magnetic resonance imaging (MRI), and western blotting (WB). **F** Graphical illustration of stereotactic injection of AAV into bilateral CA1 regions of hippocampus. **G** Representative fluorescence image of frozen brain section

In order to select the shRNA against *Sirt1* with significant knockdown efficiency, two *Sirt1*-interfering plasmids (sh-Sirt1A, sh-Sirt1B) were constructed and packaged by lentivirus. Then GL261 cells were transfected with these *Sirt1*-interfering lentiviruses, and real-time quantitative polymerase chain reaction (RT-qPCR) was performed to detect knockdown efficiency of sh-Sirt1A and sh-Sirt1B. The results showed that *Sirt1* mRNA expression levels of both GL261-shSirt1A (t_2_ = 10.29, *p* = 0.0005) and GL261-shSirt1B (t_2_ = 4.234, *p* = 0.0133) were much lower than those of the GL261-WT group, and the GL261-shSirt1A group had the lowest expression level (Fig. 1A). Therefore, the *Sirt1*-interfering plasmid with sh-Sirt1A sequence was used in subsequent experiments. Western blotting was also performed to further confirm the successful *Sirt1* knockdown in GL261 cells. The results also showed that *Sirt1* expression levels in the shSirt1 group were obviously reduced compared with the GL261-WT group (GL261-shSirt1A: t_2_ = 8.423, *p* = 0.001; GL261-shSirt1B: t_2_ = 6.106, *p* = 0.004) (Fig. 1B,C; Additional file 1: Fig. S1). For in vivo transfection, the sh-Sirt1A plasmid containing the EGFP reporter gene was used for AAV packaging, and AAV packaging the plasmid only expressing EGFP was used for sham operation (Fig. 1D).

In this study, 8-month-old C57/BL mice were randomly divided into three groups (control, sham, shSirt1) [23]. Hippocampal *Sirt1* knockdown in the shSirt1 group was conducted by stereotactic injection of AAV-CMV-shSirt1-EGFP into the dorsal hippocampal CA1 region (Fig. 1F), a brain region closely related to cognition [24]. Mice in the sham group were injected with AAV-CMV-EGFP and the mice in the control group suffered none treatment. Three weeks after injection, fluorescence imaging was performed after DAPI staining based on frozen sections of the whole brain. The obvious green fluorescence of EGFP was clearly found along the CA1 region, and there is also a slight expression of EGFP in the CA2 and CA3 regions (Fig. 1G), indicating the successful AAV transfection.

Hippocampus plays a vital role in cognition and many factors can cause its morphological changes and functional impairment, such as AD and aging [25]. So, we would like to explore the impact of *Sirt1* knockdown on hippocampal volume. Therefore, 3T brain structural MRI was performed to measure the hippocampal volume of mice 21 days after stereotactic injection to ensure sufficient *Sirt1* knockdown. Then, the mice were subjected to behavioral tests to assess cognitive changes, starting with a low-stimulating OFT, followed by a high-stimulating MWM test. There was a 3-day break between two behavior paradigms to prevent the impact of OFT on MWM results. Finally, western blotting was used to detect changes in protein levels of tau-related proteins (T-tau, P-tau at Ser396) as well as synaptic proteins (PSD95, Synaptophysin, Synapsin1, and NMDAR2B) in the hippocampus after *Sirt1* knockdown (Fig. 1E).

### *Sirt1* knockdown reduced hippocampal volume in middle-aged mice

**Fig. 2:**
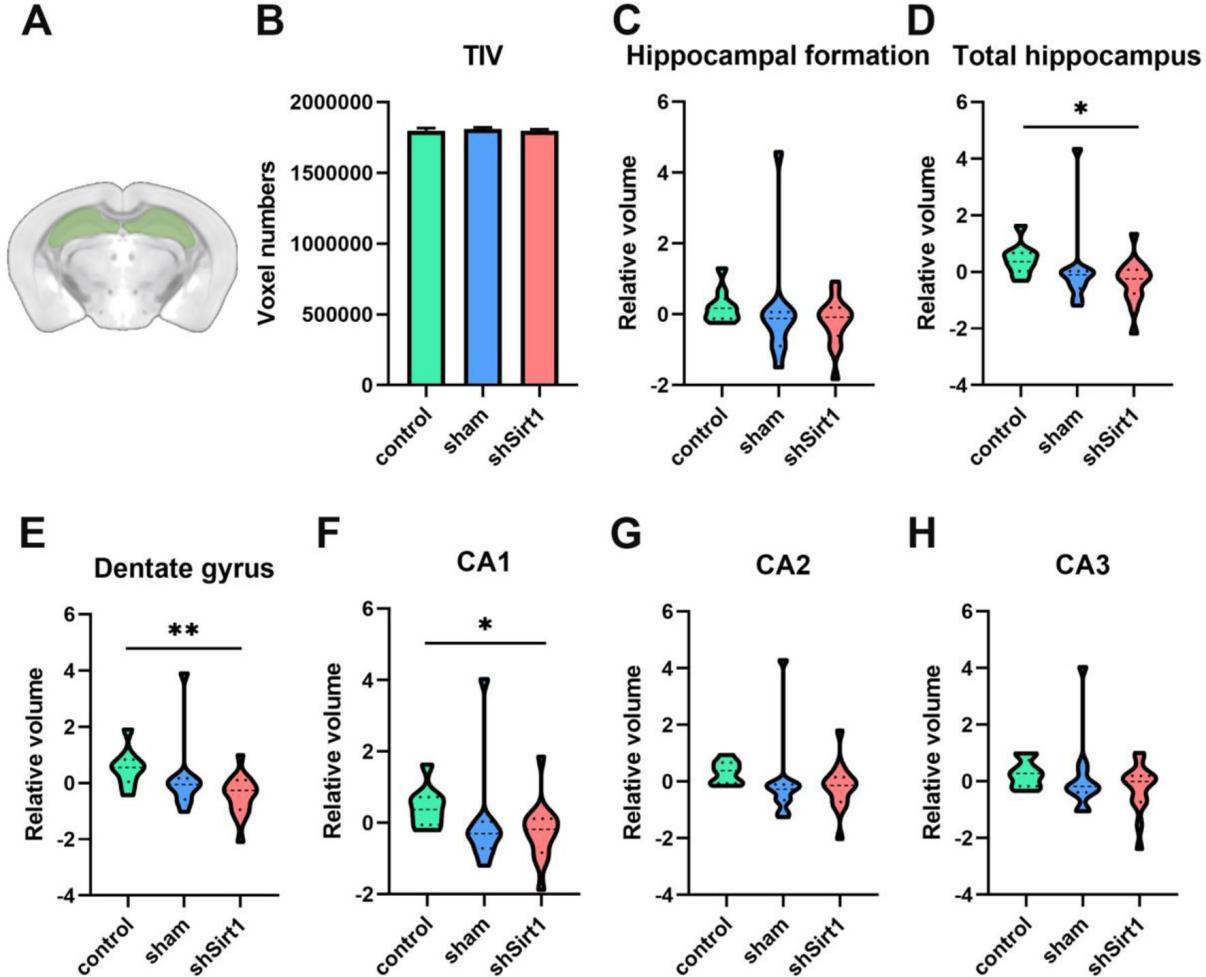
Volume analysis of hippocampus and its subregions based on 3T MR brain structural imaging. **A** Illustrative schemes for hippocampal segmentation based on Turone Mouse Brain Atlas and Template (TMBTA). **B–G** Statistical analysis of (**B**) total intracranial volume (TIV), (**C**) the volume of hippocampal formation region, (**D**) total hippocampal volume, and the volumes of hippocampal subregions including (**E**) dentate gyrus region, (**F**) CA1 region, (**G**) CA2 region, and (**H**) CA3 region. n = 9. ^*^p < 0.05, and ^**^p < 0.01 represent comparison between the control group and the shSirt1 group

3T brain structural MRI was performed on mice in three groups to detect hippocampal volume. According to TMBTA, the mice brains were segmented to measure the total intracranial volume (TIV), the volume of hippocampal formation, the total hippocampal volume, and the volumes of hippocampal subregions including dentate gyrus (DG) region, CA1 region, CA2 region and CA3 region (Fig. 2A). Results showed that there was no statistical difference in the TIV among the three groups (Fig. 2B). In the case of regression of the TIV, we performed two-sample t-test on above volumes of mice between groups. The volume of hippocampal formation in the shSirt1 group was slightly smaller compared with the control group and the sham group without statistical significance (Fig. 2C). Surprisingly, the total hippocampal volume in the shSirt1 group was significantly lower (t_2_ = 2.578, *p* = 0.0172) than that in the control group (Fig. 2D).

Further analysis of the various structures of the hippocampus revealed that the most obvious region of atrophy caused by hippocampal *Sirt1* knockdown was the DG region compared to the control group (t_2_ = 3.312, *p* = 0.0032) (Fig. 2E), followed by CA1 region (t_2_ = 2.192, *p* = 0.0392) (Fig. 2F), and there was no statistical difference in the volume of other regions (Fig. 2G, H). Compared with the control group, the total hippocampal volume and each subregion volume in the sham group showed a decreasing trend, but there was no statistical difference, which might be a slight effect caused by stereotactic injection. The reduction of hippocampal volume, also defined as hippocampal atrophy, is a well-established and validated biomarker for cognitive impairment [26, 27]. Based on our MRI results, we reasonably speculate that AAV-mediated hippocampal knockdown of *Sirt1* would cause the burden to the cognitive functions such as learning and memory in middle-aged mice.

### *Sirt1* knockdown caused cognitive impairment in middle-aged mice

**Fig. 3:**
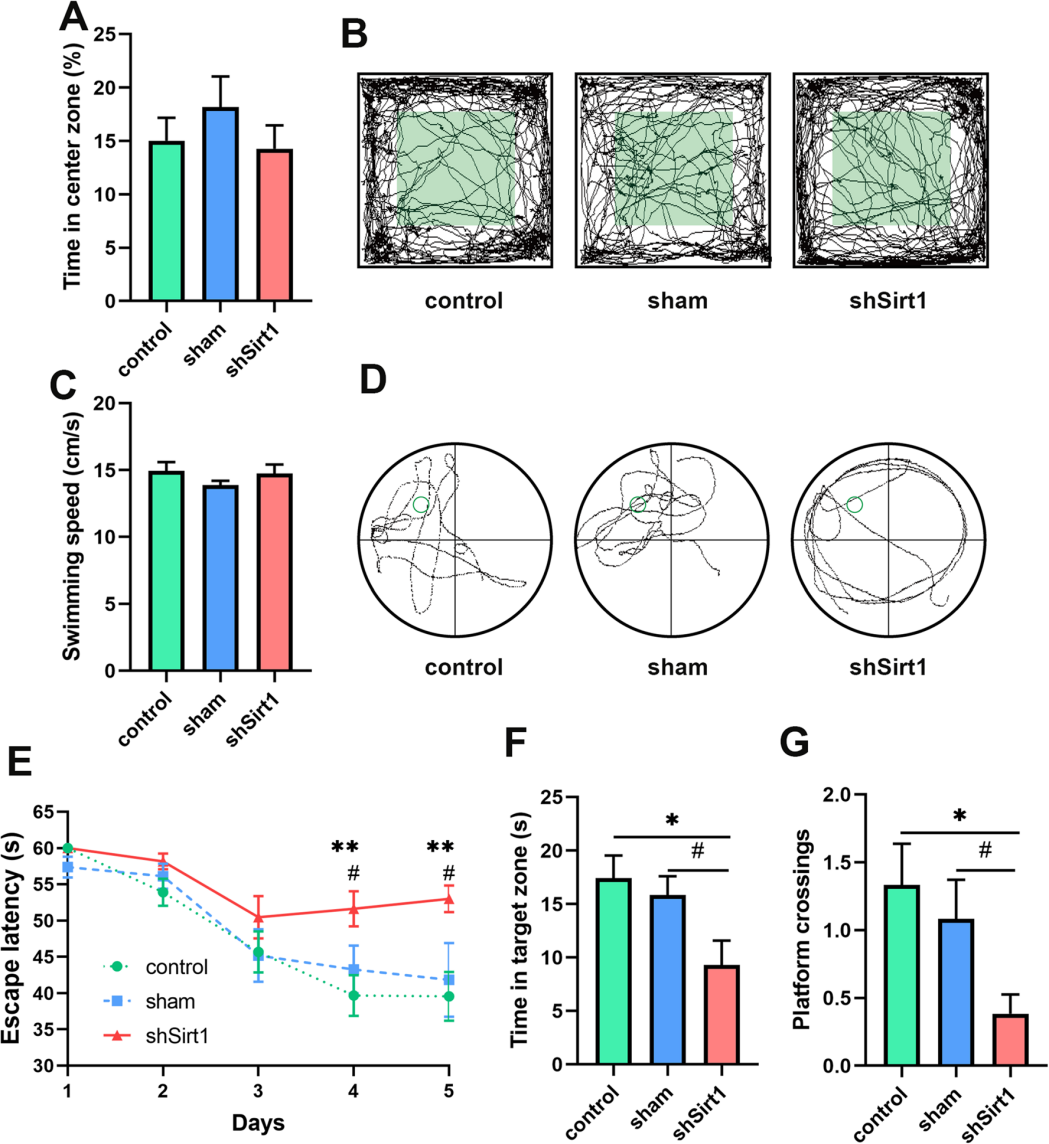
Hippocampal Sirt1 knockdown caused cognitive impairment in middle-aged mice.** A** Percentage of time spent by mice in the center zone during the OFT. **B** Representative autonomous trajectory maps of mice in OFT. Green color indicates the defined center zone and the rest is defined peripheral zone. **C** The swimming speed of mice in MWM with visual platform before the learning phase. **D** Representative swimming paths of mice in the probe phase. **E** Escape latency of mice during the learning phase. **F** Time spent in target zone and **(G)** the number of platform crossings of mice in the probe phase. n = 12. ^*^p < 0.05 and ^**^p < 0.01 represent comparison between the control group and the shSirt1 group. ^#^*p* < 0.05 represents comparison between the sham group and the shSirt1 group

To verify the adverse effects of hippocampal *Sirt1* knockdown on cognitive function, we implemented proper behavioral paradigms including OFT and MWM test. First, OFT was conducted as one of the most popular behavioral tests to assess the loco-motor activity and exploratory behavior in rodents [28]. Mice were allowed to freely explore in the experimental chamber for 15 min without any visual, auditory and olfactory disturbance, and their movements were analyzed. It was found that mice behaved similarly among three groups and preferred to spend little time (14−17%) exploring the center zone (Fig. 3A). The trajectory maps showed that mice in three groups moved mainly in the peripheral zone and occasionally moved into the center zone (Fig. 3B). Our OFT results consisted with previously reported study [23], indicating that hippocampal *Sirt1* knockdown had no effect on anxiety and exploratory behavior in middle-aged mice.

Then, MWM test was performed to assess spatial learning and memory of mice in three groups [29]. It was found that there was no statistical difference on swimming speed among three groups, indicating that all mice had normal vision and locomotor ability (Fig. 3C). During the 5-day learning phase, the escape latency of mice in the shSirt1 group was gradually prolonged, and the difference was significant from the 4th day, compared with the control group (day4: t_2_ = 3.228, *p* = 0.0032; day5: t_2_ = 3.504, *p* = 0.0016) and the sham group (day4: t_2_ = 2.096, *p* = 0.0468; day5: t_2_ = 2.313, *p* = 0.0296) (Fig. 3E). After removing the platform on the sixth day, mice in the shSirt1 group showed more chaotic swimming paths, while mice in the other two groups were more concentrated in the target zone where the platform was located (Fig. 3D). Mice in the shSirt1 group exhibited shorter time in target zones, compared to the control group (t_2_ = 2.614, *p* = 0.0142) and the sham group (t_2_ = 2.171, *p* = 0.0396) (Fig. 3F). Less number of crossings over the platform region was also found in the shSirt1 group than that in the control group (t_2_ = 2.697, *p* = 0.0121) and the sham group (t_2_ = 2.238, *p* = 0.0352) (Fig. 3G). These results revealed that conditional *Sirt1* downregulation in the hippocampus causes impairment to spatial learning and memory of middle-aged mice without effect on exploratory behavior.

### *Sirt1* knockdown can activate tau hyperphosphorylation and induce synaptic damage

**Fig. 4:**
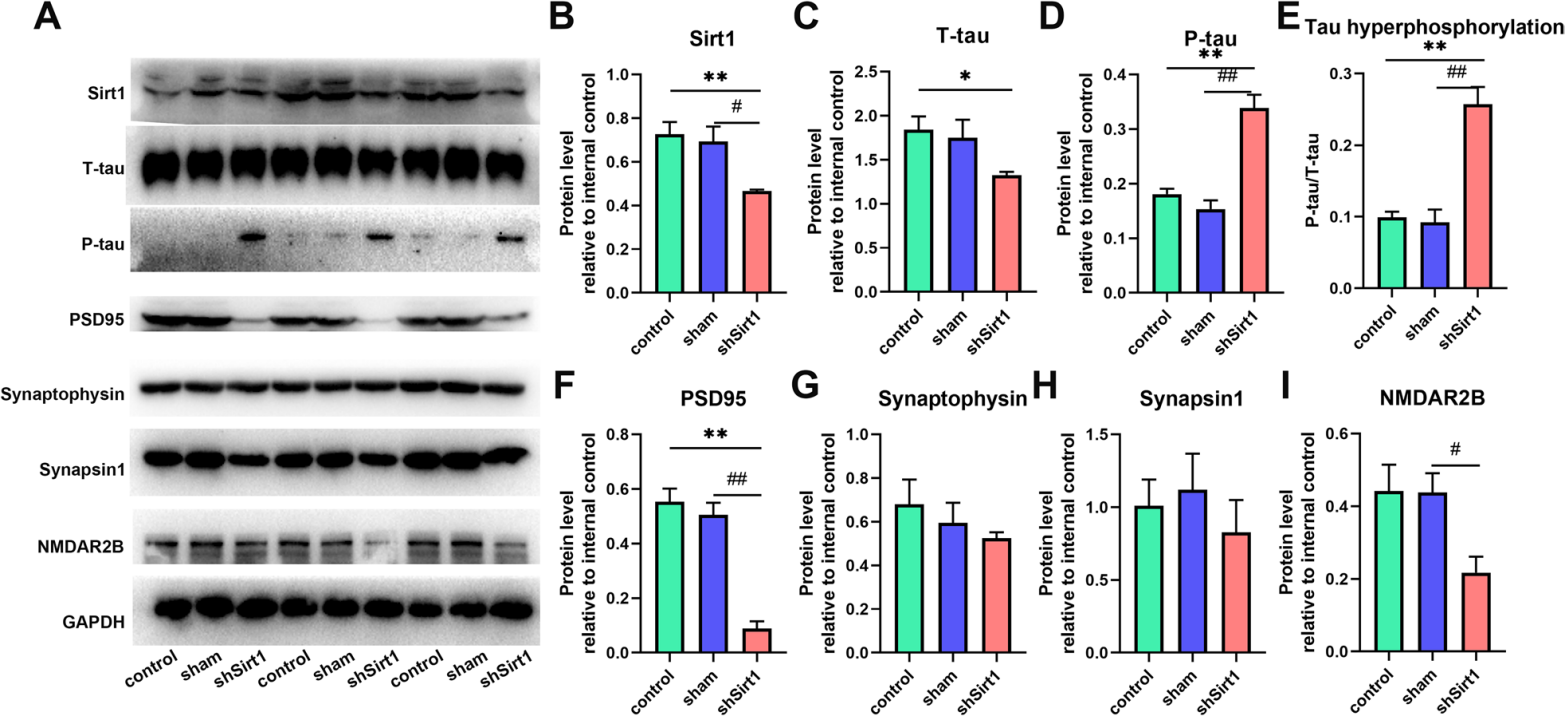
Western blotting analysis of related protein levels after hippocampal *Sirt1* knockdown.** A** Representative immunoblots. **B–I** Quantitative density values in **A**: **(B)** *Sirt1*, **(C)** T-tau, **(D)** P-tau at Ser396, **(E)** P-tau/T-tau, **(F)** PSD95, **(G)** Synaptophysin, **(H)** Synapsin1, **(I)** NMDAR2B. n = 3. ^*^p < 0.05, and ^**^p < 0.01 represent comparisons between the control group and the shSirt1 group. ^#^*p* < 0.05, and ^##^*p* < 0.01 represent comparisons between the sham group and the shSirt1 group

To further analyze underlying biological mechanisms of hippocampal *Sirt1* knockdown, the hippocampi of mice in three groups were manually dissected out and western blotting was performed to detect molecular changes. As mentioned above, *Sirt1* can contribute to P-tau clearance in AD model mice [30] and maintenance of synaptic plasticity [22]. So, the expression levels of tau-related proteins as well as synaptic proteins were detected in the shSirt1 group compared to the control group and the sham group (Fig. 4A; Additional file 1: Fig. S2). First, the significant deceased levels of *Sirt1* in the shSirt1 group (the control group: t_2_ = 4.618, *p* = 0.001; the sham group: t_2_ = 3.289, *p* = 0.030) confirmed the successful downregulation of *Sirt1* in the hippocampus (Fig. 4B). It was found that total tau (T-tau) levels decreased slightly in the shSirt1 group (the control group: t_2_ = 3.287, *p* = 0.030) (Fig. 4 C). As expected, the levels of P-tau at Ser396, as one of the important pathological features and biomarkers of AD [31], were significantly upregulated in the shSirt1 group compared to the control group (t_2_ = 6.020, *p* = 0.004) and the sham group (t_2_ = 6.400, *p* = 0.003) (Fig. 4D). The percentage of P-tau/T-tau more accurately demonstrated the effect of *Sirt1* knockdown on activating tau hyperphosphorylation (the control group: t_2_ = 6.247, *p* = 0.003; the sham group: t_2_ = 5.488, *p* < 0.005) (Fig. 4E).

To more fully assess the effect of *Sirt1* knockdown on synaptic function, we examined the expressions of four important synaptic proteins. The levels of PSD95, a postsynaptic protein regulating maturation of synapses and maintaining normal synaptic functions [32, 33], was significantly reduced in the shSirt1 group compared to the control group (t_2_ = 8.555, *p* = 0.001) and the sham group (t_2_ = 8.029, *p* = 0.001) (Fig. 4F). The levels of Synaptophysin, the most abundant membrane protein of synaptic vesicles involved in exo-endocytosis of synaptic vesicles [34, 35], and Synapsin1, a vital member of synaptic family proteins involved in the release of neurotransmitters [36, 37] showed a very slight downregulation and no significant difference was found among the three groups (Fig. 4G,H). And NMDAR2B, a subunit of NMDA receptor that regulates synaptic plasticity and learning and memory processes [38–40], were significantly decreased in the shSrit1 group (the sham group: t_2_ = 3.186, *p* = 0.033) (Fig. 4I). These results revealed that hippocampal *Sirt1* knockdown can activate tau hyperphosphorylation and induce synaptic damage in the hippocampus of middle-aged mice.

## Discussion

*Sirt1* is the first identified nicotinamide-adenine dinucleotide (NAD+)-dependent HDAC [41], and it regulates various biological processes such as cellular senescence [42], AD [43], cancer [44] and neuroinflammation [45]. Studies have shown that *Sirt1* knockout (*Sirt1*-KO) would cause cognitive impairment and defects in synaptic plasticity, but the brains of *Sirt1*-KO mice exhibited normal morphology [22]. Since the hippocampus is the main brain area for cognition and its volume is an important neuroimaging phenotype for clinical evaluation of AD [25, 46], assessing hippocampal volume is more convincing for cognition evaluation than assessing changes in whole brain structure. Furthermore, the protective effect of *Sirt1* on hippocampal volume in aged mice has not been reported. Therefore, we conducted conditional hippocampal *Sirt1* knockdown by stereotactic injection of AAV expressing *Sirt1* shRNA. In vivo knockdown efficacy of *Sirt1* shRNA was confirmed by fluorescence staining of frozen mouse brain sections and western blotting analysis. 3T brain structural MRI was performed to investigate the volume changes of hippocampus and its subregions caused by AAV-mediated hippocampal *Sirt1* knockdown in middle-aged mice. Combined with OFT, MWM test and western blotting, we found that *Sirt1* knockdown induced hippocampal atrophy was also accompanied by cognitive impairment, activation of hippocampal tau hyperphosphorylation and synaptic damage.

shRNA is a widely used tool for gene knockdown with high specificity [47], and lentivirus and AAV are the most commonly used vector tools for in vitro and in vivo genetic modification, respectively [48, 49]. Firstly, two *Sirt1*-shRNA plasmids were designed and packaged as lentiviruses respectively to transfect G1261 cells. RT-qPCR based on the extracted RNA was used to detect the knockdown efficiency and the *Sirt1*-shRNA plasmid with relatively highest knockdown efficiency was confirmed by western blotting and selected for subsequent AAV packaging. For in vivo studies, 8-month-old mice were used because they are widely considered to represent a healthy middle-aged mouse model. AAV expressing *Sirt1*-shRNA was injected into bilateral CA1 of dorsal hippocampus by stereotactic injection, and 3T brain structural MRI was performed on the mice three weeks later. The results showed that in the case of regression of the TIV, the volumes of total hippocampus, DG and CA1 regions were significantly reduced in the shSirt1 group compared to the control group, while the volumes of hippocampal formation (hippocampus and parahippocampal area [50]), CA2 and CA3 regions were not significantly changed. The volumes of hippocampus and its subregions were slightly decreased in the sham group compared to the control group, which might be caused by stereotactic injection itself. In addition, fluorescence imaging of frozen brain sections showed green fluorescence in the CA2 and CA3 regions, whereas MRI results showed no significant changes in the volumes of these two regions, which may be due to insufficient expression of shSirt1 carried by AAV migrating from the injection site. The DG region without obvious green fluorescence had obvious volume changes, which may be due to the impact of other biological processes caused by *Sirt1* knockdown on the volume of the hippocampus, which deserves further research in the future. These MRI results showed that *Sirt1* knockdown resulted in a significant decrease in hippocampal volume.

To assess hippocampus-mediated cognitive function, the classical behavioral paradigms, OFT and MWM test, were successively used to evaluate anxiety, exploratory activity and spatial learning and memory of mice. The lowly stimulating OFT was performed first, taking 15 min per mouse; Then the mice rested for three days to prevent the influence between the two behavioral paradigms; The highly stimulating MWM was finally performed, with each mouse undergoing a five-day learning phase and a one-day exploration phase, for a total of six days. In OFT, there were no statistical difference in trajectory map and time spent in the center zone among the three groups. Besides, time mice spent in the center zone in our study consisted with previously reported studies [23, 51]. OFT results indicated that *Sirt1* knockdown in the hippocampus had no effect on anxiety and exploratory activity in middle-aged mice. However, in the MWM test, the control group and the sham group showed similar purposeful swimming, while the shSirt1 group performed very poorly. MWM results indicated that *Sirt1* knockdown in the hippocampus prolonged escape latency, reduced time spent in target zone and number of platform crossing, and seriously impaired the spatial learning and memory ability in middle-aged mice.

Finally, hippocampal tissues of the three groups were randomly isolated for western blotting to examine the potential mechanisms of *Sirt1* knockdown in regulating hippocampal atrophy and cognitive impairment. The significant decrease of *Sirt1* levels confirmed the successful *Sirt1* knockdown in mouse hippocampus. Tau phosphorylation at serine 396 epitope is strongly implicated in AD-associated tau pathology [52], therefore, the significant increase of P-tau levels at Ser396 suggested that *Sirt1* knockdown could activate tau hyperphosphorylation in mouse hippocampus. The obvious downregulation of PSD95 and NMDAR2B levels in the shSirt1 group showed the vital role of *Sirt1* in maintaining synaptic integrity and function.

## Conclusions

Taken together, hippocampal *Sirt1* knockdown could lead to hippocampal atrophy and its induced cognitive impairment in middle-aged mice, along with activation of tau hyperphosphorylation and synaptic damage. This work revealed the key role of *Sirt1* in maintaining hippocampal volume to prevent cognitive impairment during aging, and provides important targets for the prevention and therapy of AD.

## Materials and methods

### Construction and packaging of *Sirt1* interference plasmid

According to the design principles of shRNA and the nucleotide sequence of *Sirt1* gene in GenBank (NM_019812.3), two *Sirt1* shRNA sequences were designed. Forward and reverse oligoes of sh-Sirt1A as follows: CCGGCGCGGATAGGTCCATATACTTCTCGAGAAGTATATGGACCTATCCGCGTTTTTG;AATTCAAAAACGCGGATAGGTCCATATACTTCTCGAGAAGTATATGGACCTATCCGCG; Forward and reverse oligoes of sh-Sirt1B as follows: CCGGGCCATGAAGTATGACAAAGATCTCGAGATCTTTGTCATACTTCATGGCTTTTTG;AATTCAAAAAGCCATGAAGTATGACAAAGATCTCGAGATCTTTGTCATACTTCATGGC. The synthesized single-stranded oligonucleotides were annealed to form double-stranded DNA, and then ligated with plko.1 by restriction enzyme BshTI/EcoRI. Then the competent bacterium DH5a was transformed and a single colony was selected and sequenced. The colonies with correct sequencing results were amplified to extract the target plasmids.

We transfected the target plasmids, together with lentivirus vectors PAX8 and VSVG, into HEK293T cells for lentivirus packaging. The knockdown efficiency of obtained *Sirt1* interference lentiviruses were verified by Quantitative PCR in Mouse glioma cells GL261. Briefly, GL261 cells were transfected with lentiviruses for 1 week. Then, total RNA was extracted from cells with a TRIzol reagent (Gibco, 15,596,018) according to the manufacturer’s instructions. Then RNA was reverse transcribed into cDNA with an RT-PCR kit (Accurate Biology, AG11705). Quantitative real-time RT-PCR (RT-qPCR) was carried out on a Mx3005p real-time polymerase chain-reaction system (Agilent Technologies, USA) using ChamQ Universal SYBR qPCR Master Mix (Vazyme, Q311-02) and the temperature was set as follows: initial denaturation for 1 min at 95 °C, followed by 40 cycles of 15 s at 95 °C, 20 s at 58 °C, and 45 s at 72 °C. The PCR primers were designed as follows: forward, 5-GTGGCAGTAACAGTGACAGTGG-3; reverse, 5-TCCAGATCCTCCAGCACATTCG-3. To examine the expression of *Sirt1* protein, GL261 cells after transfection were collected and lysed by RIPA lysis buffer mixed with Phenylmethylsulphonyl fluoride (PMSF) (Solarbio LIFE SCIENCES, P0100) and a Phosphatase inhibitor cocktail A (Beyotime, p1081) and cellular proteins were subjected to western blotting.

The Sirt1 mRNA expression was normalized comparing to rpo. For transfection in vivo, the target plasmids were packaged with AAV by Lianyungang ChuangRui Biological Product Trading Company Ltd. (Jiangsu, China). The final titer of *Sirt1* knockdown AAV (AAV-CMV-shSirt1-EGFP) and the no-load control AAV (AAV-CMV-EGFP) was 7.1 × 10^12^ vp/mL and 3.5 × 10^12^ vp/mL, respectively.

### Animals

C57BL/6J mice (8 months old, male, 35–40 g) were purchased from Beijing HFK Bioscience Co. Ltd. (Beijing, China). The mice were used for experiments at least 14 days after acclimatization to laboratory conditions. The mice were placed in polycarbonate cages with 3–5 mice per cage at a controlled temperature (22 ± 1 °C) for 12-h light-dark cycle and *ad libitum* access to food and water. All animal experiments were performed in accordance to *Animal Research: Reporting of In Vivo Experiments* (ARRIVE guidelines) [53] and the guidelines of Institutional Animal Care and Use Committee at Tianjin Medical University (IACUC number E2015093) and following reported protocols [54, 55].

### Hippocampal *Sirt1* knockdown in aged mice

The mice were randomly divided into three groups, each consisting of 14–16 mice: the untreated mice (control), the mice injected with AAV-CMV-EGFP (sham), and the mice injected with AAV-CMV-shSirt1-EGFP (shSirt1). The mice were anesthetized with inhalation of 2% isoflurane throughout the process by a small animal anesthesia machine (R510-22, RWD Life Science Co., Ltd., China) and fixed on a stereotactic apparatus (G1124701, RWD Life Science Co., Ltd., China). Both AAV-CMV-EGFP and AAV-shSirt1-EGFP were diluted to 3.5 × 10^12^ vp/mL. Bilateral injection with 1 µL of above AAV was performed into the dorsal hippocampal CA1 region, and stereotaxic coordinates were shown as follows: AP–2.00 mm, ± ML 1.5 mm, DV -1.0 mm from bregma. The injection rate was controlled at 100 nL/min. The needle syringe was left in place for about 10 min before being withdrawn. The scalp was sutured, disinfected with iodophor, and the mice were kept warm. After awakening from anesthesia, they were put back into the cage. After three weeks, the construction of *Sirt1* knockdown in mouse hippocampus was considered successful [56].

### Fluorescence staining of frozen mouse brain sections

Mice were anesthetized with 3% isoflurane and executed by cervical dislocation. Then, mice were subjected to heart perfusion with 4% paraformaldehyde (Biosharp, China) to fix mouse brain tissue. Next, the whole brains were isolated properly and fixed in paraformaldehyde overnight. After dehydration in 30% sucrose solution (30% m/v sucrose in PBS) for 2 days, the brains were embedded into optimal cutting temperature compound (OCT) (Sakura, Japan) and frozen in – 80 °C for 1 day. The brains were sectioned into 20 μm slices at – 22 °C. The slices were collected on adhesion microscope slides (CITOTEST Scientific, China) and stained with DAPI. Finally, processed slices were observed and filmed by an Olympus IX73 inverted microscope (Japan).

### 3T brain structural MRI

The mice were anesthetized 15 min MRI scanning by intraperitoneal injection with 4% chloral hydrate at 0.2 mL/10 g. Then the mice were fixed on a semi-circular small animal scanning frame, their limbs were fixed with medical tape, and their heads were fixed by hanging a thin wire through the incisors. MRI was performed on a 3T MRI scanner (DISCOVERY MR750, General Electric, USA) with a mouse brain coil. The parameters for 3D T_1_-weighted fast acquisition of the whole mouse brain were as follows: repetition time (TR) = 12.6 ms, echo time (TE) = 6.0 ms, field of view (FOV) = 3.0 × 1.0 mm, slice thickness = 0.3 mm, number of slices = 1746, frequency = 180, phase = 150, prep time = 500 ms, flip angle = 12°, bandwidth = 15.63, locs per slab = 128, number of excitations = 4, and scan time = 20 min 9 s. For voxel-based morphometry (VBM) analysis, obtained MR DICOM files were subjected to conversion to NIFTI files using dcm2niix, augmentation of the voxel size 14 times using DPABI [57], automatic segmentation of hippocampus based on Turone Mouse Brain Atlas and Template (TMBTA) using SPM12 software [58]. TMBTA defined mouse hippocampus into 7 subregions, including hippocampal formation, CA1 field, CA2 field, CA3 field, molecular layer of Dentate gyrus, polymorph layer of Dentate gyrus and granule cell layer of Dentate gyrus. We summed the volume of molecular layer of Dentate gyrus, polymorph layer of Dentate gyrus and granule cell layer of Dentate gyrus to measure total Dentate gyrus and summed all the 7 subregions to measure total hippocampus. After the regression of the total intracranial volume, the two-sample t-test was used to analyze the difference in gray matter volume between the three groups within the hippocampus.

### Open field test

Open field test (OFT) is a common animal behavior experiment to detect the loco-motor activity and exploratory behavior of mice. The open field apparatus (RWD Life Science Co., Ltd., China) consisted of a square arena (50 × 50 cm) with walls 45 cm high. The arena was divided into the center area (30 cm × 30 cm square) and the peripheral area. The mice arrived at the test site 24 h in advance to ensure that they were acclimated to the environment, and the mice were stroked for 1–2 min to reduce non-specific stress stimulation. Each mouse was gently and quickly placed in the central area with their backs to the experimenter, and the experimenter immediately left. The SMART3.0 digital tracking system (Panlab, USA) automatically recorded the movements of mice in the arena. The exploring time of each mouse was 15 min, and the proportion of time spent in the central area was measured.

### Morris water maze test

The Morris Water Maze (MWM) test is a classical behavioral task to test hippocampal-dependent learning and memory of mice, consisting of 5 days of learning phase and 1 day of probe phase. Room and water temperature were maintained at 22 °C. A circular tank (120 cm diameter, 50 cm height) was divided into four quadrants with distinctive landmarks as visual cues, and equipped with a hidden platform (8 cm diameter, – 1 cm below the water surface). Before the test, the platform was lifted 1 cm above the water surface, and the mouse was released to swim freely at the furthest site from the platform. The swimming speeds were recorded by the equipped SMART 3.0 Video Tracking System (Panlab, USA). Every day during learning phase, the mouse was released from each quadrant and swam for 60 s. Once the mouse found the platform within 60 s and stayed on it for 3 s, the system automatically recorded this period as escape latency. If the mouse did not find the platform within the 60 s, the system recorded escape latency as 60 s. The experimenter guided the mouse to the platform and allowed it to stay there for 10 s. On the sixth day, the platform was removed, the mouse was released at the furthest site from the platform and allowed to freely explore for 60 s. During probe phase, the swimming paths, the time spent in target quadrant, and the numbers of mice crossing the platform location were also recorded.

### Western blotting

Mouse hippocampal tissues were harvested, cut with ophthalmic scissors, and lysed with RIPA lysis buffer mixed with Phenylmethylsulphonyl fluoride (PMSF) (Solarbio LIFE SCIENCES, P0100) and Phosphatase inhibitor cocktail A (Beyotime, p1081). The proteins were separated by 10–12% SDS-polyacrylamide gel electrophoresis (SDS-PAGE) and transferred to polyvinylidene fluoride (PVDF) membrane (Merck, Ireland). Then, the membranes were blocked with 5% non-fat milk for 1 h at room temperature. The membranes cut into proper bands following by incubated with primary antibodies overnight at 4 °C. The primary antibodies included SIRT1 Polyclonal antibody (Proteintech, 13161-1-AP), Phospho-Tau (Ser396) Recombinant Polyclonal Antibody (5HCLC) (Invitrogen, 710,298), anti-Tau (Abcam, ab80579), PSD95-Specific,DLG4 Polyclonal Antibody (Proteintech, 20,665), Anti-NMDAR2B (Abcam, ab254356), SYN1-Specific Polyclonal antibody (Proteintech, 20258-1-AP) and Recombinant Anti-Synaptophysin antibody (Abcam, ab32127). After washing three times with PBST, the membranes were incubated with Goat Anti-Rabbit IgG H&L (HRP) (Abcam, ab205718) for 1 h at room temperature. β-Tubulin and GAPDH were used as internal controls. After washing three times with PBST, immunoreactive bands were visualized using enhanced chemi-luminescence (ECL) (NCM Biotech, P10300) detection regent, and the film was taken by a ChemiDoc XRS + System (Biorad, USA). The densitometric analysis of band intensities was carried out using the Image J software (National Institutes of Health, Bethesda, MD, USA).

### Statistical analysis

All data were analyzed with SPSS R23.0.0.0 software. Data were expressed as mean ± standard error (*SEM*). Statistical comparisons between experimental group and control group or sham group were performed by using two-tailed unpaired Student’s test. *p* < 0.05 was considered statistically significant.

## Supplementary Information

**Additional** **file** **1:** **FigureS1. **Original blots of *Sirt1* levels after *Sirt1* knockdown in GL261 cells. **FigureS2. **Original blots of related protein levels after hippocampal *Sirt1 *knockdown.

## Data Availability

Data generated during this study are included in this published article and supplementary files. The mRNA data are also available in the Harvard Dataverse repository (10.7910/DVN/MXNIGT). The raw MRI datasets generated during the current study are not publicly available due to privacy and ethical restrictions, but are available from the corresponding author upon reasonable request.
