# Supplementary material for: Sirt1 protects against hippocampal atrophy and its induced cognitive impairment in middle-aged mice
Source: BMC Neurosci. 2022 Jun 6;23:33. doi: 10.1186/s12868-022-00718-8 (PMC9169381; doi:10.1186/s12868-022-00718-8)
Supplement: Supplementary file 1 — Additional file 1: FigureS1. Original blots of Sirt1 levels after Sirt1 knockdown in GL261 cells. FigureS2. Original blots of related protein levels after hippocampal Sirt1 knockdown. [file 12868_2022_718_MOESM1_ESM.docx]

Additional file

for

***Sirt1* protects against hippocampal atrophy and its induced cognitive impairment in middle-aged mice**

Zuhao Sun^1,2†^, Shuang Zhao^1†^, Xinjun Suo^1,2^, Yan Dou^1^*

*^1^ Department of Radiology and Tianjin Key Laboratory of Functional Imaging, Tianjin Medical University General Hospital, Tianjin 300052, P. R. China*

*^2^ School of Medical Technology, Tianjin Medical University, Tianjin 300070, P. R. China*

^†^These authors contributed equally to this work.

***Correspondence:** douyan@tmu.edu.cn (Y. D.)


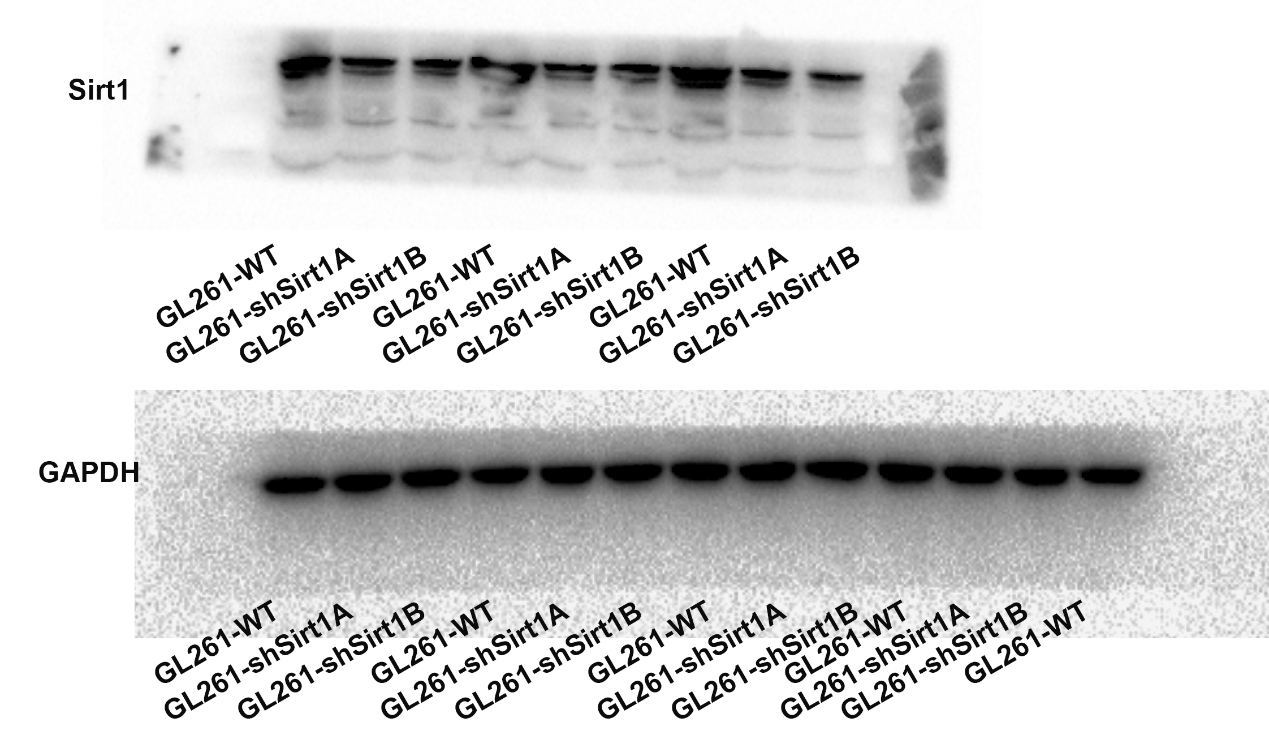


**Figure S1.** Original blots of *Sirt1* levels after *Sirt1* knockdown in GL261 cells.


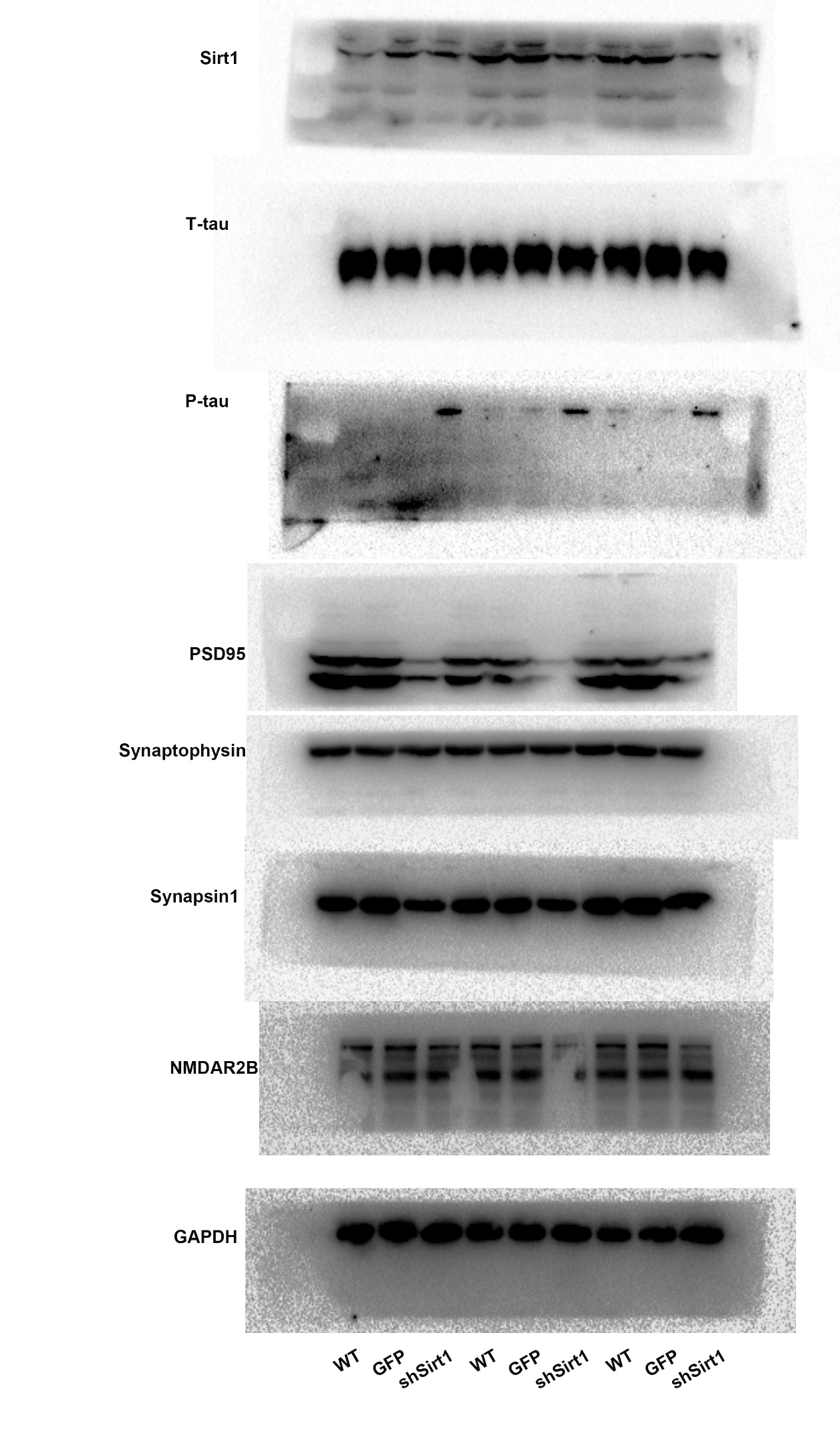


**Figure S2.** Original blots of related protein levels after hippocampal *Sirt1* knockdown.
